# Supplementary material for: Influence of transurethral enucleation with bipolar of the prostate on erectile function: Prospective analysis of 51 patients at 12-month follow-up
Source: PLoS One. 2022 Aug 11;17(8):e0272652. doi: 10.1371/journal.pone.0272652 (PMC9371260; doi:10.1371/journal.pone.0272652)
Supplement: Attachment — Submitted filename: Response to Reviewers.docx [file pone.0272652.s003.docx]

**Replies to the Editor and Reviewers**

Dear Dr. Kobayashi,

Thank you for submitting your manuscript to PLOS ONE. After careful consideration, we feel that it has merit but does not fully meet PLOS ONE’s publication criteria as it currently stands. Therefore, we invite you to submit a revised version of the manuscript that addresses the points raised during the review process.

ACADEMIC EDITOR:

I hope you can adapt your manuscript as the reviewers requested. My advice is also to write your conclusin in a more modest way.

1→Thank you very much for your suggestion. I changed the sentence to make it easier to understand. We revised the text to reflect this change (Conclusion section, page 29, line 398).

Please ensure that your manuscript meets PLOS ONE's style requirements, including those for file naming.

2→Thank you very much for your advice.

I change the file name as follows:

Fig. 1→Fig1

We combined 6 figures into 1 figure in Fig. 2 and added panels e and f to panels a-d.

Although not pointed out, is it possible to make the following corrections?

3→We deleted the abbreviation “n.s.: not significant” in Table 2 and now show the actual p values.

Upon re-submitting your revised manuscript, please upload your study’s minimal underlying data set as either Supporting Information files

4→Thank you very much for your advice.

I upload our study’s minimal underlying data set as a Supporting Information file (file name: S2_Table).

Your ethics statement should only appear in the Methods section of your manuscript.

5→Thank you very much for your advice.

I deleted the “Ethics approval and consent to participate” section.

Reviewer #1: I read the manuscript entitled:" Influence of transurethral enucleation with bipolar of the prostate on erectile function: Prospective analysis of 51 patients at 12-month follow-up" All parts were written well but there is a concern about selection bias. All patients had erectile dysfunction before surgery (moderate as a whole or mild to moderate in group a and even severe in group b). It is probable if the authors select patients with good erectile function maybe they could find any effect on erectile function.

6→Thank you very much for your comments and suggestion. We classified the patients according to their preoperative IIEF-5 score into subgroup a: severe (IIEF-5 5-7, n=23), subgroup b: moderate (IIEF-5 8-11, n=13), subgroup c: mild to moderate (IIEF-5 12-16, n=9), subgroup d: mild (IIEF-5 17-21, n=5), and subgroup e: no ED (IIEF-5 22-25, n=1). There was a slight but nonsignificant decrease in the IIEF-5 score in the comparison of preoperative, 3-, and 12-month follow-up data in subgroup c (14, 13, 11) but no remarkable changes in subgroups d (18, 16, 18) and e (23, 23, 23). Although we cannot say for sure because of the small number of patients, we have to consider the possibility of worsening of EF in subgroup c but we infer that the influence of TUEB on EF is minor in subgroups d and e. We revised the text to reflect these results (Abstract section, page 2, line 31; Material and methods section, page 9, lines 146, 152; Results section, page 15, line 203/page 16, lines 209, 225; and Discussion section, page 23, line 292/page 27, lines 356, 358/page 28, line 376) and revised Fig 2 and Table 4.

Reviewer #2: The authors must be congratulated for their intent to evaluate the influence of TUEB on EF in patients with BPO. However, the study shows all the results of a prospective database. Therefore, either the objective must be changed, or the results have to be adjusted, focusing in the proposed objective. That being said, I would recommend to devide the patients according to the previously described ED classification based on the IIEF-5 score: severe (5-7), moderate (8-11), mild to moderate (12-16), mild (17-21), and no ED (22-25).

7→Thank you very much for your suggestion. We classified the patients according to your valuable advice as follows: subgroup a: severe (IIEF-5 5-7, n=23), subgroup b: moderate (IIEF-5 8-11, n=13), subgroup c: mild to moderate (IIEF-5 12-16, n=9), subgroup d: mild (IIEF-5 17-21, n=5), and subgroup e: no ED (IIEF-5 22-25, n=1). There was a slight but significant increase in the IIEF-5 score in the comparison of preoperative and 12-month follow-up data in subgroup a. There was a slight but nonsignificant decrease in the IIEF-5 score in the comparison of preoperative, 3-, and 12-month follow-up data in subgroup c (14, 13, 11) but no remarkable changes in subgroups d (18, 16, 18) and e (23, 23, 23). Although we cannot say for sure because of the small number of patients, we have to consider the possibility of worsening of EF in subgroup c but we infer that the influence of TUEB on EF is minor in subgroups d and e. We revised the text to reflect these results (Abstract section, page 2, line 31/page 3, line 45; Material and methods section, page 9, lines 146, 152; Results section, page 15, lines 203, 206/page 16, lines 209, 210; and Discussion section, page 23, lines 292, 294/page 26, line 341/page 27, lines 356, 357, 358/page 28, line 376) and revised Fig 2 and Table 4.

Reviewer #3: 1. Are you comparing your data to HoLEP and enucleation techniques or all prostate reducing procedures?

8→Thank you very much for your question. We compared our data with previously reported results of transurethral enucleation surgery such as HoLEP and ThuLEP. Although Qmax at 12-month follow-up was slightly low and the rate of urinary tract infection was relatively high, we think our data were almost comparable with those of previous studies. We revised the text to reflect these results (Discussion section, page 21, lines 252, 258/page 22, lines 264, 268, 269) and added Supplementary Table 1.

2. Advantages of using Bipolar enucleation versus well established enucleation techniques such as HoLEP and ThuLEP?

9→Thank you very much for your question. We think TUEB is relatively easy to learn, and we are able to control intraoperative bleeding with ease. We also think that we can precisely enucleate the adenoma from the capsule, which might reduce injury to the sphincter. We revised the text to reflect your suggestion (Discussion section, page 20, line 241).

3. The data needs to be evaluated against TURP and thus a randomized prospective trial between the two would be necessary since the size of the gland appears to be in TURP category.

10→Thank you very much for your suggestion. We compared our data with a prospective study between TUEB and TURP. Although Qmax at 12-month follow-up was slightly low, we think our data were almost comparable with those of this study. We revised the text to reflect this result (Discussion section, page 21, lines 255, 258) and added Supplementary Table 1.

4. Many limitations to your study including one institution, size limitation, no comparison to other modalities, and too many patients with ED before the surgery.

11→Thank you very much for your comment. We revised the limitations to reflect your suggestion (Discussion section, page 28, lines 376, 381, 384).

5. Bipolar enucleation has not been established against TURP or other enucleation procedure.

12→Thank you very much for your comment. We revised the text to reflect your suggestion (Introduction section, page 4, line 65).

6. Long catheterizations post op is concerning.

13→Thank you very much for your comment. We think that we might be able to remove the catheter earlier. A previous study reported that TUEB required a shorter catheterization time. We revised the text to reflect your suggestion (Discussion section, page 27, line 368).

By the way, in the discussion, ureteral catheterization should be changed to urethral catheterization.

14→Thank you very much for pointing out our mistake. We corrected this error (Discussion section, page 28. line 370).

7. The big advantage of enucleation techniques are for large glands usually over 100 grams, all the prostates in this study were less. What was the impetus for bipolar enucleation in this study?

15→Thank you very much for your comment and question. This study included 10 patients with prostate size over 100 mL, and we were able to perform surgery without any problems using the same procedure. We revised the text to reflect your suggestion (Discussion section, page 28, line 384).

8 I would suggest a re-write and compare your data only to enucleation procedures from a previously published randomized prospective trial. The flow the paper should be better and more concise. Your goal appears to suggest Bipolar enucleation can be performed with minimal effects on ED and with good outlet outcomes.

16→Thank you very much for your comment. As mentioned, we compared our data with a randomized prospective trial of transurethral enucleation surgery. Although Qmax at 12-month follow-up was slightly low and the rate of urinary tract infection was relatively high, we think our data were almost comparable with these studies. We might be able to suggest that TUEB can be performed with minimal effects on ED and with good outlet outcomes. We revised the text to reflect this result (Discussion section, page 21, lines 252/258; page 22, lines 264/268, 269) and added Supplementary Table 1.

Word limit in the Abstract section

17→Dear editor,

I revised the Abstract section according to the comments of Reviewers #1 and #2. As a result, the word count now exceeds 300 words (304 words). I hope this will not be a problem.

While revising your submission, please upload your figure files to the Preflight Analysis and Conversion Engine (PACE) digital diagnostic tool

→Thank you very much for your advice. I checked the figure files with PACE.
