# Supplementary material for: Influence of transurethral enucleation with bipolar of the prostate on erectile function: Prospective analysis of 51 patients at 12-month follow-up
Source: PLoS One. 2022 Aug 11;17(8):e0272652. doi: 10.1371/journal.pone.0272652 (PMC9371260; doi:10.1371/journal.pone.0272652)
Supplement: Attachment — Submitted filename: Response to Reviewers.docx [file pone.0272652.s004.docx]

**Replies to the Editor and Reviewers**

Dear Dr. Kobayashi,

Thank you for submitting your manuscript to PLOS ONE. After careful consideration, we feel that it has merit but does not fully meet PLOS ONE’s publication criteria as it currently stands. Therefore, we invite you to submit a revised version of the manuscript that addresses the points raised during the review process.

ACADEMIC EDITOR:

Can you revise again, with help of the reviewers comments?

→We are grateful for this additional opportunity to revise our paper. We thank you and the reviewers for the time and effort spent providing fine feedback to strengthen our paper. We have revised and submitted our updated manuscript.

Journal Requirements:

Please review your reference list to ensure that it is complete and correct. If you have cited papers that have been retracted, please include the rationale for doing so in the manuscript text, or remove these references and replace them with relevant current references. Any changes to the reference list should be mentioned in the rebuttal letter that accompanies your revised manuscript. If you need to cite a retracted article, indicate the article’s retracted status in the References list and also include a citation and full reference for the retraction notice.

→Thank you very much for your suggestion. We have checked all articles cited in the references, and none show any indication that they have been retracted.

Reviewers' comments:

Reviewer's Responses to Questions

Reviewer #2: Thanks for addressing all the raised questions.

Some minor issues:

Abstract:

Material and methods:

The groups identification is quite complex. There is initially a GROUP A and GROUP B. Then, there are subgroups a, b, c, d and e. This leads to misunderstandings and is hard to interpretate. Maybe you could use groups 1 and 2 and subgroups a to e.

1→Thank you very much for your valuable advice, which has made the results easier to understand. We revised the two main group names to Group 1 and Group 2 throughout the manuscript and in Tables 1 and 4.

I could not understand the following sentence: “Data are displayed as median or median (interquartile range) or (preoperative, at 3-month follow-up, at 12-month follow-up).”. In the abstract and in the method section.

2→Thank you very much for your advice. We deleted the confusing second half of the sentence to make it easier to understand. We revised the text to reflect this change in the Abstract, page 2, line 33, and the Material and Methods section, page 9, line 149.

Methods

Line 142: Please describe the groups in a new paragraph

3→Thank you very much for your suggestion. We revised the text to reflect your suggestion (Material and Methods section, page 8, line 143).

Line 138: statistical analysis description should be the last paragraph of the methods section.

4→Thank you very much for your suggestion. All text related to statistical analysis is included in the last paragraph of the Material and Methods section (page 9, lines 149-161).

Results/ Objectives:

Once again, I believe the results and discussion deal with a lot of things that are not mentioned in the objective of the study!

The aim of the study was: “Therefore, we conducted a single-center, prospective study to evaluate the influence of TUEB on EF in 51 patients with BPO.”

The following topics have nothing to do with the aim of the study, unless you evaluate if theses complications could interfere with the post-operative erectile function.

Line 172: correlation between TZ volume and resection weight

5→Thank you very much for your advice, which has improved the flow of this paper and made it more concise. We deleted the sentence you pointed out and another related sentence (Material and Methods section, page 9, line 152 and Results section, page 13, line 179).

Line 175: patients with transient urinary retention after catheter removal, urinary retention resolved spontaneously without oral administration of medication

6→We deleted the sentence you pointed out (Results section, page 13, line 181).

Line: 177: patients with hematuria

7→We deleted the sentence you pointed out (Results section, page 13, line 183).

Line 179: urethral structure and bladder neck sclerosis

8→We deleted the sentence you pointed out (Results section, page 13, line 185).

Table 3 is enough to show the complications due to the procedure.

Figure 1 is not necessary.

9→Thank you. We deleted Figure 1. Previous Figure 2 is now Figure 1.

Discussion:

Regarding complications, instead of citing 7 studies, I would cite

1) for incontinence (line 254):

- Castellani D, Rubilotta E, Fabiani A, Maggi M, Wroclawski M, Teoh JYC, Pirola GM, Gubbioti M, Pavia MP, Gomez Sancha F, Galosi AB, Gauhar V. Correlation between transurethral interventions and their influence on type and duration of postoperative urinary incontinence: results from a systematic review and meta-analysis of comparative studies. J Endourol. 2022 May 19. doi: 10.1089/end.2022.0222.

10→Thank you for alerting us to these three very useful papers. We revised the text to reflect your suggestions (Discussion section, page 22, line 279).

2) For urethral stricture (line 255):

- Pirola GM, Castellani D, Lim EJ, Wroclawski ML, Le Quy Nguyen D, Gubbiotti M, Rubilotta E, Chan VW, Corrales M, Rojo EG, Herrmann TRW, Teoh JY, Gauhar V. Urethral stricture following endoscopic prostate surgery: a systematic review and meta-analysis of prospective, randomized trials. World J Urol. 2022 Feb 13. doi: 10.1007/s00345-022-03946-z.

11→We revised the text to reflect your suggestion (Discussion section, page 22, line 281).

3) For bladder neck sclerosis (line 255):

- Castellani D, Wroclawski ML, Pirola GM, Gauhar V, Rubilotta E, Chan VW, Cheng BK, Gubbiotti M, Galosi AB, Herrmann TRW, Teoh JY. Bladder neck stenosis after transurethral prostate surgery: a systematic review and meta-analysis. World J Urol. 2021 Nov;39(11):4073-4083. doi: 10.1007/s00345-021-03718-1.

12→We revised the text to reflect your suggestion (Discussion section, page 23, line 282).

Lines 259 – 268 (TZ volume and MRI) does not correlate to the topic of the study. In my opinion, it should be deleted.

13→We deleted the related sentences you pointed out (Discussion section, page 23, lines 286-295).

Line 365: Regarding the safety and efficacy of TUEB, I would cite the metanalysis that compared laser versus non-laser enucleation procedures and did not find any significant differences between the techniques (Wroclawski ML, Teles SB, Amaral BS, Kayano PP, Cha JD, Carneiro A, Alfer W Jr, Monteiro J Jr, Gil AO, Lemos GC. A systematic review and meta-analysis of the safety and efficacy of endoscopic enucleation and non-enucleation procedures for benign prostatic enlargement. World J Urol. 2020 Jul;38(7):1663-1684. doi: 10.1007/s00345-019-02968-4.)

14→Thank you for telling us about this very useful paper. We revised the text to reflect your suggestion (Discussion section, page 29, lines 394-396).

Line 375: Enucleation is a procedure indicated for prostates of any size according to the AUA guidelines and for prostates over 30g according to the EAU guidelines. So, no need to justify your approach.

15→Thank you very much for your advice. We deleted the sentences to reflect your suggestion (Discussion section, page 29, lines 398-400).

Reviewer #3: This is one section that should be revised.

"HoLEP is a well-established enucleation surgery in patients with BOO

233 [5]. However, HoLEP requires much experience and appropriate endoscopic

234 skills to avoid complications [21], whereas TUEB is relatively easy to learn

235 [7]. We can coagulate any bleeding immediately during adenoma enucleation

236 because the TUEB loop is equipped with a standard wire loop. In addition, we

237 can precisely enucleate the adenoma from the capsule with the TUEB loop,

238 which might reduce injury to the sphincter at the apex [11]."

This section should be re-written. This is truly an opinion from the surgeons doing bipolar enucleations. Learning curve has been defined with other procedures over longer time period at different institutions. Reproducibility is an important part of learning curve and multi-institutional trials need to be done to appropriately comment on this.

Phrasing should be not so presumptive. Accuracy with Holmium and Thulium of bleeders are also easily accomplished with experience. I realize your enthusiasm of this technique, however, you need to tone it down throughout the discussion and introduction. Maybe try the phrase, "In our experience,..." to phrase some the presumptive statements. In addition, please focus on erectile function as the title would suggest.

16→Thank you very much for your advice. We used the phrase "In our experience" to focus on the ease of learning TUEB and reduced any presumptiveness on our part. We also focused on erectile function in this paragraph (Discussion section, page 21, lines 251-259).
